# Molecular epidemiology of blastocystosis in Malaysia: does seasonal variation play an important role in determining the distribution and risk factors of *Blastocystis* subtype infections in the Aboriginal community?

**DOI:** 10.1186/s13071-017-2294-2

**Published:** 2017-07-31

**Authors:** Samseh Abdullah Noradilah, Norhayati Moktar, Tengku Shahrul Anuar, Ii Li Lee, Fatmah Md Salleh, Siti Nor Azreen Abdul Manap, Noor Shazleen Husnie Mohd Mohtar, Syed Muhamad Azrul, Wan Omar Abdullah, Anisah Nordin, Syamsa Rizal Abdullah

**Affiliations:** 1 Department of Parasitology and Medical Entomology, Faculty of Medicine, Universiti Kebangsaan Malaysia Medical Centre, Cheras, Kuala Lumpur, Malaysia; 2 Department of Medical Sciences II, Faculty of Medicine and Health Sciences, Universiti Sains Islam Malaysia, Pandan Indah, 55100 Kuala Lumpur, Malaysia; 3 Department of Pre-Clinical Sciences, Faculty of Medicine and Health Sciences, Universiti Tunku Abdul Rahman, Sungai Long Campus, Selangor, Malaysia; 4 Integrative Pharmacogenomics Institute, Universiti Teknologi MARA, Puncak Alam Campus, Selangor, Malaysia; 5 Centre of Medical Laboratory Technology, Faculty of Health Sciences, Universiti Teknologi MARA, Puncak Alam Campus, Selangor, Malaysia; 6 Kulliyyah of Medicine and Health Sciences, Kolej Universiti INSANIAH, Kuala Ketil, Kedah Malaysia; 7 Multipurpose Laboratory, Faculty of Medicine, Universiti Kebangsaan Malaysia Medical Centre, Cheras, Kuala Lumpur, Malaysia

**Keywords:** *Blastocystis*, Seasonal variations, Risk factors, Person to person transmission, Water-borne transmission

## Abstract

**Background:**

Alternating wet and dry seasons may play an important role in the acquisition and distribution of *Blastocystis* subtype infection in the tropics. This cross-sectional study was therefore conducted to provide the prevalence of *Blastocystis* and to determine the potential risk factors associated with each subtype during the wet and dry seasons in the Aboriginal community, Pahang, Malaysia.

**Methods:**

A total of 473 faecal samples were collected: 256 (54.1%) and 217 (45.9%) samples were obtained during the wet (October-November 2014) and the dry season (June 2015), respectively. All fresh faecal samples were subjected to molecular analysis for subtype and allele identification.

**Results:**

Of the 473 samples, 42.6% and 37.8% were positive for *Blastocystis* ST1, ST2, ST3 and ST4 during wet and dry seasons, respectively. Prevalence of *Blastocystis* ST1 was significantly higher during the wet season compared to the dry season (*Z* = 2.146, *P* < 0.05). Analysis of the association of each *Blastocystis* subtype with socioeconomic characteristics showed the presence of other family members infected with *Blastocystis* ST3 and the use of stored river water for domestic activities were the significant risk factors for *Blastocystis* ST3 infections during both seasons. Untreated water supply and low monthly household income (less or equal to RM 500) were the other significant risk factors for *Blastocystis* ST3 infections during wet and dry season, respectively. The presence of other family members with *Blastocystis* ST1 and ST2 was the only significant risk factor associated with ST1 and ST2 infections during both seasons. We hypothesise that transmission of *Blastocystis* ST1, ST2 and ST3 occurred from person to person during both seasons. The waterborne transmission was also identified as a mode of transmission of *Blastocystis* ST3.

**Conclusion:**

The significant risk factors identified in this study were important in the dynamic transmission of *Blastocystis* infections during both seasons. Provision of treated water supply and health education are affirmative actions to be taken to control *Blastocystis* infections in this community.

**Electronic supplementary material:**

The online version of this article (doi:10.1186/s13071-017-2294-2) contains supplementary material, which is available to authorized users.

## Background

*Blastocystis* is one of the most commonly detected group of anaerobic parasites in the human intestine [1, 2]. Infection with *Blastocystis* leads to gastrointestinal symptoms which include acute or chronic diarrhoea, abdominal pain, nausea, vomiting, anorexia pruritus and tenesmus [3]. There are many studies carried out in Asia, Australia, Europe and America to determine the prevalence of *Blastocystis* in humans, animals or drinking water [4]. Interestingly, *Blastocystis* has been included in WHO Guidelines for Drinking-water Quality [5]. This parasite has been detected in the tap water (12%) of premises of infected patients [6]. It has also been associated with a number of outbreaks in the United Kingdom [7, 8]. *Blastocystis* has been identified microscopically in the river and tap water samples in Iran (13.63%; 6/44) [9]. Moreover, *Blastocystis* ST3 was detected in wastewater and reuse water in Mexico [10]. Individuals who consumed boiled water are less likely to acquire *Blastocystis* infection in comparison to those who do not [11]. Other than water, humans have been incriminated to be a source for *Blastocystis* transmission. Anuar et al. [12] reported human to human transmission among family members through multivariate analysis. A significant association was established between orphans and the infected childcare workers suggesting person-to-person transmission of *Blastocystis* [13].

Although many researchers successfully isolated *Blastocystis* spp. from humans and there are numerous studies highlighting several risk factors associated with transmission of *Blastocystis*, information on the potential risk factors associated with each subtype of *Blastocystis* infection during wet and dry seasons in the tropics are still lacking. In addition, no previously published studies in Malaysia has ever included data on the alleles of *Blastocystis*. Differences of exposure in humans not reflected at subtype level can be identified at the allele level [14]. To the best of our knowledge, this is the first study on the prevalence and potential risk factors associated with *Blastocystis* subtypes infections during two seasons in Malaysia. Identification of the seasonal variation in the distribution, prevalence and risk factors of *Blastocystis* subtypes infections is a public health priority. Understanding the impact of different seasons on blastocystosis is crucial in mitigating the transmission of the disease. It is hoped that the finding of this study will benefit the Aboriginal communities and related authorities to initiate prevention and control program against *Blastocystis* infection.

## Methods

### Study area and sampling

**Fig. 1:**
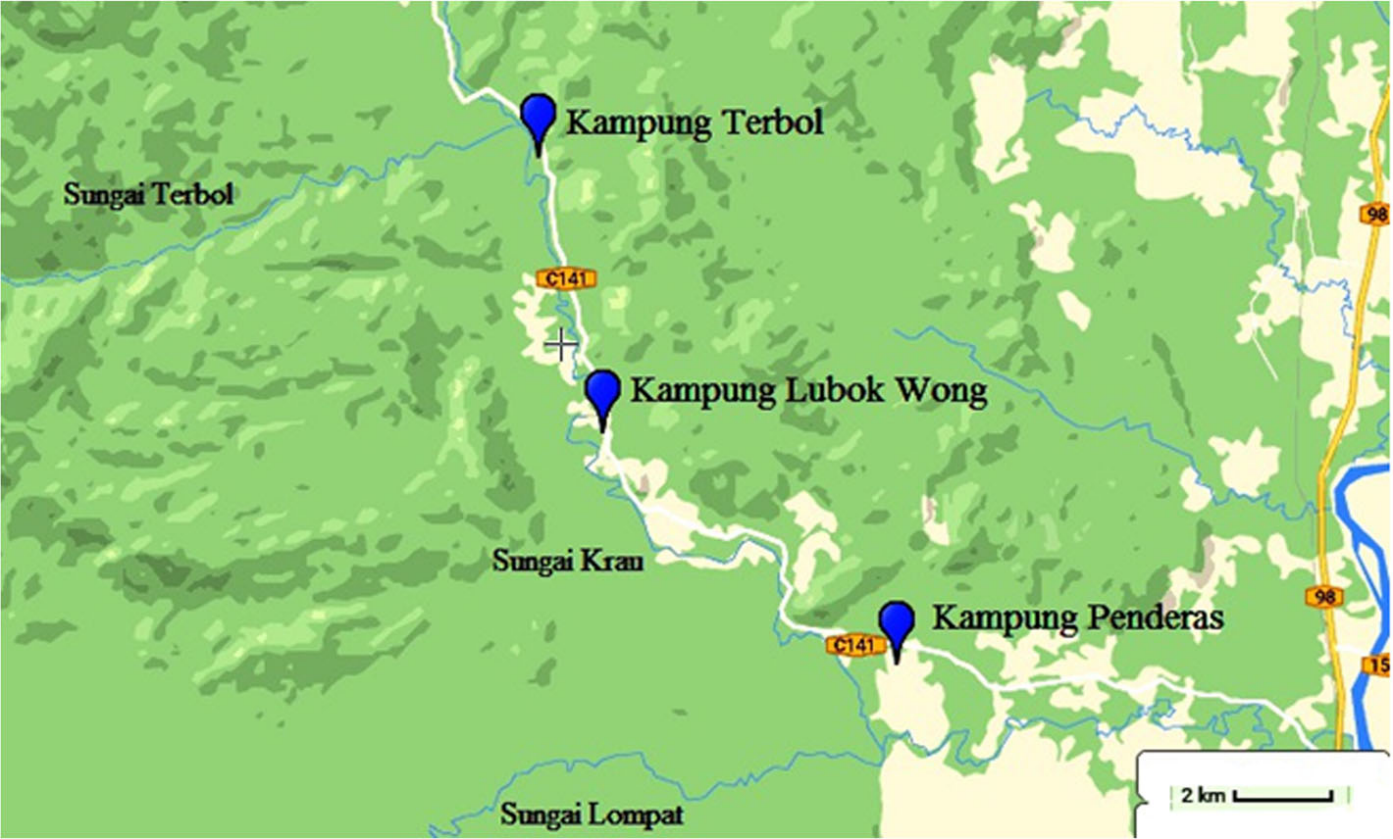
Map showing the three Aboriginal villages in the study

The cross-sectional study were carried out during two seasons (wet season: October–November 2014) and dry season (June 2015) among 473 subjects living in three Aboriginal settlements in Kuala Krau, Temerloh, Pahang, Malaysia; Kampung Terbol (3°48′50.08″N, 102°13′48.11″E), Kampung Lubok Wong (3°46′2.75″N, 102°14′27.38″E) and Kampung Penderas (3°43′38.93″N, 102°17′26.02″E) (Fig. 1). Samples were collected by a randomised sampling of the Aborigines residing in the villages located upstream, midstream and downstream from Sungai Krau. All village entry was approved by the Ministry of Rural and Regional Department of Malaysia. The sample size was calculated based on the formula published by Kish [15]. With an expected prevalence of *Blastocystis* at 24.7% [12] and 52.3% [16] at the confidence interval of 95% and an absolute precision of 0.05, the appropriate sample size for the study was estimated to be 343–460 subjects. Within each village, those who provided consent to participate were included in this study. Exclusion criteria included refusal to participate (i.e. refuse to give consent, failed to submit stool samples or absent during the parasitological survey).

### Structured questionnaires

To determine the risk factors and outcomes of *Blastocystis* infection, a structured pre-tested questionnaire on demographic and socio-economic data, behavioural risks, source and treatment method of drinking water, animal contacts, sanitation and living conditions [12] was included. The structured questionnaire consists of (i) demographic data (i.e. age, gender and education level); (ii) socioeconomic background (i.e. occupation, household income and educational status); (iii) behavioural risks (i.e. personal hygiene such as hand washing); (iv) environmental sanitation and living conditions (i.e. types of water supply, latrine system, sewage disposal system and the presence of domestic animals) [12]. In the case of child participants, parents or guardians who gave informed consent were interviewed on behalf of their children.

### Collection of faecal samples

Following the administration of the questionnaire, wide-mouth, screw-capped containers pre-labelled with the individuals’ names and codes were distributed to all subjects for the collection of their faecal samples (*c.*10 mg) the next day. The faecal container was kept in a zip-locked plastic bag. The fresh faecal samples were brought to the Community Laboratory, Department of Parasitology and Medical Entomology, Faculty of Medicine, Universiti Kebangsaan Malaysia Medical Centre.

### Genomic DNA extraction, amplification and sequencing

All faecal samples were subjected to genomic DNA extraction using QIAamp® Fast DNA Stool Mini Kit (QIAGEN, Hilden, Germany), as per the manufacturer’s instructions. Screening of the samples was performed using the primers BhRDr (5′-GAG CTT TTT AAC TGC AAC AAC G-3′) and RD5 (5′-ATC TGG TTG ATC CTG CCA GT-3′) [17]. PCR amplification with 25 mM MgCI_2_, 10 mM dNTP and 5 units/μl of Taq Polymerase, was performed using an Eppendorf Pro-S thermal cycler (Hamburg, Germany) with a final volume of 30 μl and included 30 cycles of initial denaturation at 95 °C for 5 min, followed by denaturation at 95 °C for 1 min, annealing at 63.3 °C for 1 min and 30 s, extension at 72 °C for 1 min and an additional cycle of 10 min at 72 °C. The PCR products were separated on a 1.5% agarose gel at 90 V (Major Science, California) for 80 min and visualised by ultraviolet light illumination using a GelDoc UV Transilluminator (BioRad, Hercules, USA). Genomic DNA from *Blastocystis*-positive samples was sent to Genomics Bioscience Taiwan for sequencing. The sequences obtained were compared with sequences available in the GenBank database using BLASTn (http://www.ncbi.nlm.nih.gov/BLAST). The sequences were deposited in GenBank under the accession numbers KX351975–KX351997. Identification of alleles was performed by sequence query into *Blastocystis* subtype (18S) and Sequence Typing (MLST) databases at *https://pubmlst.org/﻿blastocystis/* [14].

### Phylogenetic analysis

Positive *Blastocystis* sequences and reference sequences (ST1-ST4) obtained from GenBank were aligned using ClustalX 2.0 [18], and a phylogenetic tree was constructed with the best-fit model using the Maximum Likelihood method under the Kimura two-parameter (K2P) model [19] using the Mega 7.0.14 software [20]. Bootstrap analyses of 1000 replicates were performed to identify the measure of support for the generated clades. *Proteromonas lacertae* was used as the outgroup.

### Statistical analysis

Data obtained from the questionnaire and laboratory procedures were entered into the Statistical Package for Social Sciences software for Windows (SPSS Version 23, Chicago, IL, USA). A Chi-square (*χ* ^2^) test was used to identify the associations between the variables. In the univariate analysis, the dependent variable was the prevalence of *Blastocystis* subtypes while the independent variables were demographic and socioeconomic factors, behavioural risks, environmental sanitation and living condition characteristics and gastrointestinal symptoms. All factors that were significant in the univariate model were included in a logistical multivariate regression analysis to identify the most significant risk factors. The level of statistical significance was set at *P* < 0.05. Odds ratio (OR) and 95% confidence interval (CI) were computed for both univariate and multivariate logistical regression analyses for each statistically significant factor. A significant difference in the prevalence of *Blastocystis* subtypes between wet and dry seasons was analysed using proportionate test with a level of significance set at *P* < 0.05.

## Results

### Characteristics of the study population

A total of 473 faecal samples were collected from Aboriginal community members aged 2 to 81 years old. Two-hundred and fifty-six (54.1%) samples were obtained during the wet season and 217 (45.9%) were obtained during the dry season. With regard to the age groups, ﻿211 (44.6%)﻿ were less than 15 years old while 262 (55.4%) were 15 years old or more. Subjects who participated in this study comprised 228 (48.2%) males and 245 (﻿51.8%) ﻿(51.2%) females; 139 (54.3%) females and 117 (45.7%) males participated in this study during the wet season and 106 (48.8%) females and 111 (51.2%) males during the dry season. Approximately 126 (34.4%) of the fathers had no formal education while 114 (31.1%) of the mothers have no formal education.

The majority of the subjects worked as farmers and rubber tappers (82.5%; 390/473) and earned a monthly income of less than RM 500 (66.8%; 316/473). A total of 31.7% of the Aborigines had household family members of more than eight people. More than half of the studied subjects used untreated tap water supply (63.6%) and river water (50.5%) for their daily use. About 43.3% of the Aborigines have no proper latrine system and practice open defecation in the river and bushes. Animals including dogs, chickens, ducks, and cats were reared by 275 (58.1%) Aboriginal participants.

### Prevalence of blastocystosis between wet and dry seasons according to subtypes

Table 1 shows the distribution of *Blastocystis* subtypes. Of 473 faecal samples 63 (13.3%), 27 (5.7%), 98 (20.7%) and 3 (0.6%) were positive for *Blastocystis* infection ST1, ST2, ST3 and ST4, respectively, with an overall prevalence of 40.4%. With regard to season, 42.6% of the subjects were infected with *Blastocystis* ST1 (16.4%), ST2 (6.6%), ST3 (18.8%) and ST4 (0.8%) during the wet season and and 37.8% of the subjects were infected with *Blastocystis* ST1 (9.7%), ST2 (4.6%), ST3 (23.0%) and ST4 (0.5%) during the dry season. However, the overall prevalence was not significantly different between the two seasons (*Z* = 1.058, *P* > 0.05). Similarly, there was no significant difference in the prevalences of *Blastocystis* ST2 (*Z* = 0.949, *P* > 0.05), ST3 (*Z* = 1.148, *P* > 0.05) and ST4 (*Z* = 0.437, *P* > 0.05) observed during the wet and dry seasons. However, the prevalence of the subjects infected with *Blastocystis* ST1 was significantly greater during the wet season as compared to dry season (*Z* = 2.146, *P* = 0.032). *Blastocystis* ST3 was the predominant subtype isolated throughout the wet and dry season, while ST4 was the least common subtype observed in the communities.

**Table 1:** Prevalence and significant differences of *Blastocystis* subtype infections in the wet and dry seasons

*Blastocystis* subtype	Wet season (*n* = 256)		Dry season (*n* = 217)		Significant difference between both seasons	
	No. of positive samples	Prevalence (%)	No. of positive samples	Prevalence (%)	*Z*-score	*P*-value
ST1	42	16.4	21	9.7	2.146	0.032*
ST2	17	6.6	10	4.6	0.949	0.435
ST3	48	18.8	50	23.0	1.148	0.250
ST4	2	0.8	1	0.5	0.437	0.333
Total prevalence (%)	109 (42.6%)		82 (37.8%)		1.058	0.289

*P < 0.05

### Phylogenetic tree of samples positive for *Blastocystis*

**Fig. 2:**
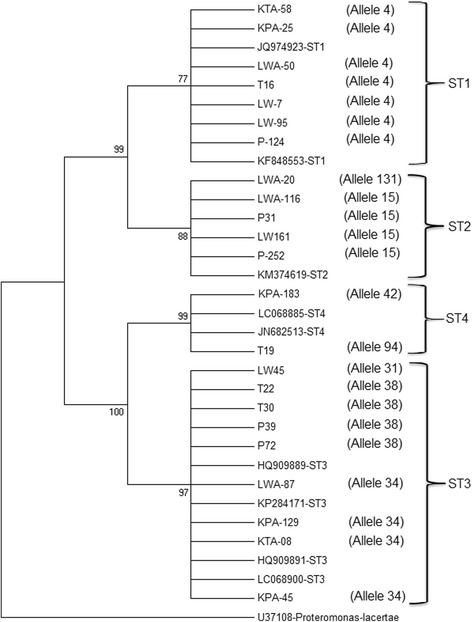
Maximum Likelihood phylogenetic tree of the positive faecal samples

Maximum Likelihood phylogeny of *Blastocystis* isolated from the faecal samples in both seasons is shown in Fig. 2 with four clades which correspond to *Blastocystis* ST1, ST2, ST3 and ST4. Twenty-three samples which were submitted to the GenBank were included in the phylogenetic analysis to represent all *Blastocystis*-positive samples. Each clade of ST3 and ST4 was supported by relatively high bootstrap value (97–99%), while ST1 and ST2 was each supported by a moderate bootstrap value (77–88%). *Blastocystis* ST1 and ST2 clustered together and shared a common ancestor. Meanwhile, ST3 and ST4 were closely related to each other in 100% of the bootstrap replications.

### Associated factors for *Blastocystis* subtype infections

Univariate analysis for all *Blastocystis* ST1-ST4 and the socioeconomic characteristics revealed significant associations of *Blastocystis* infections and the presence of other family members infected with *Blastocystis*, use of untreated tap water supply, use of untreated tap water for washing, use of stored river water for domestic use, and the absence of a latrine system during the wet season. Meanwhile, the presence of other family members infected with a similar *Blastocystis* subtype, a monthly household income of less or equal to RM 500, and use of stored river water in containers for domestic activities were found to be the significant risk factors for *Blastocystis* infections among the Aborigines during the dry season.

Logistic regression analysis confirmed that presence of other family members infected with *Blastocystis* subtypes (OR = 3.194, 95% CI: 1.808–5.644, *P* < 0.001) and use of river water stored in containers for domestic activities (OR = 2.534, 95% CI: 1.417–4.530, *P* = 0.002) were the significant risk factors in the acquisition of *Blastocystis* infections during the wet season. Meanwhile, during the dry season, presence of other family members infected with *Blastocystis* subtypes (OR = 4.731, 95% CI: 2.466–9.078, *P* < 0.001), monthly household income of ≤ RM 500 (OR = 5.313, 95% CI: 1.913–14.754, *P =* 0.001) and the use of stored river water for domestic activities (OR = 5.233, 95% CI: 2.063–13.269, *P <* 0.001) were the significant risk factors for *Blastocystis* ST1-S4 infections in the community studied.

The association of each *Blastocystis* subtype (ST1, ST2, ST3 and ST4) infection with demographic and socioeconomic factors, behavioural risks, environmental sanitation and living conditions characteristics during wet season are shown in Additional file 1: Table S1. The results show that age of less or equal to 15 years old (OR = 0.431, 95% CI: 0.210–0.887, *P* = 0.020), presence of other family members infected with ST1 (OR = 5.170, 95% CI: 2.535–10.544, *P* < 0.001) and occupation as rubber tapper and farmer, (OR = 1.181, 95% CI: 0 .043–0.755, *P* = 0.009) were significantly associated with *Blastocystis* ST1 infections. Meanwhile, *Blastocystis* ST2 infection was significantly associated with an age less or equal to 15 years old (OR = 3.169, 95% CI: 1.082–9.278, *P* = 0.028), female (OR = 1.326, 95% CI: 1.112–2.956, *P =* 0.033), presence of other family members infected with *Blastocytis* ST2 (OR = 3.077, 95% CI: 1.057–8.545, *P* < 0.001) and not washing hands after having contact with soils or gardening (OR = 3.567, 95% CI: 1.161–10.959, *P* = 0.019). *Blastocystis* ST3 infection was mainly associated with the presence of other family members infected with ST3 (OR = 5.229, 95% CI: 2.571–10.634, *P* = 0.001), performing routine activities including drinking, cooking and bathing using untreated water supply from rivers and wells (OR = 3.254, 95% CI: 1.450–7.303, *P* = 0.003), use of untreated tap water supply for washing (OR = 2.523, 95% CI: 1.921–5.341, *P* = 0.013), use of stored river water for domestic activities (washing, bathing, drinking, cooking) (OR = 2.571, 95% CI: 1.329–4.974, *P* = 0.004) and not washing hand after defaecation (OR = 3.159, 95% CI: 1.067–9.351, *P* = 0.030).

A significant parameter observed in the univariate analysis of *Blastocystis* subtypes identified during the wet season was having other family members infected with the same *Blastocystis* subtype. In addition, this was the significant risk factor to acquire each *Blastocystis* ST1, ST2 and ST3 infection; ST1 (OR = 5.632, 95% CI: 2.687–11.803, *P <* 0.001), ST2 (OR = 3.240, 95% CI: 2.339–10.449, *P <* 0.001) and ST3 infections (OR = 4.088, 95% CI: 1.907–8.762, *P* < 0.001) using logistic regression analysis. The use of untreated tap water supply for drinking, cooking and bathing (OR = 2.103, 95% CI: 1.204–3.672, *P* = 0.037) and use of stored river water for domestic activities were identified as the most significant risk factors to acquire *Blastocystis* ST3 infections (OR = 2.418, 95% CI: 1.129–5.179, *P* = 0.023) (Table 2).

**Table 2:** Multivariate analysis of the potential risk factors of *Blastocystis* subtypes infections among the Aborigines in Temerloh, Pahang, Malaysia during the wet and dry seasons

Variable	Wet season						Dry season					
	ST1		ST2		ST3		ST1		ST2		ST3	
	OR (95% CI)	*P*-value	OR (95% CI)	*P*-value	OR (95% CI)	*P*-value	OR (95% CI)	*P*-value	OR (95% CI)	*P*-value	OR (95% CI)	*P*-value
Age (≤15)	0.606 (0.257–1.429)	0.252	2.199 (0.621–7.789)	0.222	–	–	–	–	–	–	–	–
Gender (Female)	–	–	0.287 (0.888–0.942)	0.440	–	–	–	–	–	–	–	–
Occupation (Rubber tapper and farmer)	0.254 (0.052–1.244)	0.091	–	–	–	–	–	–	–	–	–	–
Presence of other family members with same *Blastocystis* subtype infection	5.632 (2.687–11.803)	<0.001^**^	3.240 (2.339–10.449)	<0.001^**^	4.088 (1.907–8.762)	<0.001^**^	9.837 (2.241–13.179)	0.002^**^	5.559 (1.904–6.213)	0.020^*^	9.814 (1.976–8.664)	<0.001^**^
Monthly household income of ≤ RM 500	–	–	–	–	–	–	–	–	–	–	2.122 (1.034–4.354)	0.038^*^
Water supply (Untreated water)	–	–	–	–	2.103 (1.204–3.672)	0.037^*^	–	–	–	–	–	–
Source of washing (Untreated tap water)	–	–	–	–	1.137 (0.900–2.097)	0.153	–	–	–	–	–	–
Usage of stored river water	–	–	–	–	2.418 (1.129–5.179)	0.023^*^	–	–	–	–	8.069 (3.425–19.011)	<0.001^**^
Not washing hands after having contact with soil	–	–	3.569 (0.723–7.610)	0.118	–	–	–	–	–	–	–	–
Did not wash hand after defaecation	–	–	–	–	2.687 (0.590–2.236)	0.201	–	–	–	–	–	–

^*^ *P* < 0.05, ^**^ *P* < 0.01

During the dry season, *Blastocystis* ST1, ST2 and ST3 infections were significantly associated with at least one of the variables studied (Additional file 1: Table S1). The presence of other family members infected with ST1 was the only factor associated with *Blastocystis* ST1 infections (OR = 4.500, 95% CI: 1.534–13.198, *P* = 0.003). Having other family members with *Blastocystis* ST2 infections was the only significant variable associated with ST2 infections (OR = 2.688, 95% CI: 1.018–9.770, *P* = 0.001). Meanwhile, the risk factors significantly associated with *Blastocystis* ST3 infections were the presence of other family members infected with ST3 (OR = 13.021, 95% CI: 6.658–18.628, *P* < 0.001)*,* monthly household income of less or equal to RM 500 (OR = 2.827, 95% CI: 1.656–7.765, *P =* 0.029) and use of stored river water for domestic activities (OR = 4.962, 95% CI: 2.476–9.946, *P* < 0.001).

Multivariate logistical regression model confirmed that having other family members infected with ST1 was the only significant risk factor identified for *Blastocystis* ST1 infections (OR = 9.837, 95% CI: 2.241–13.179, *P* = 0.002). Similarly, it was confirmed that presence of other family members with *Blastocystis* ST2 infections was the significant factor to acquire ST2 infections (OR = 5.559, 95% CI: 1.904–6.213, *P* = 0.020). Meanwhile, there were more than one risk factor to contract *Blastocystis* ST3 infections identified by logistic regression including the presence of other family members infected with ST3 (OR = 9.814, 95% CI: 1.976–8.664, *P* < 0.001)*,* household income of less or equal to RM 500 (OR = 2.122, 95% CI: 1.034–4.354, *P* = 0.038) and use of stored river water for domestic activities (OR = 8.069, 95% CI: 3.425–19.011, *P* < 0.001) (Table 2).

### Symptoms associated with *Blastocystis* subtype infections

Of the 191 participants positive for *Blastocystis* subtype infection during both wet and dry seasons, 37 (19.4%) were positive for a single infection of *Blastocystis* and 154 (80.6%) were positive for mixed infections. Fourteen out of 37 (37.8%) positive individuals complained of diarrhoea, 12 (32.4%) complained of abdominal pain, 16 (43.2%) had flatulence, and 3 (8.1%) had nausea. Univariate analysis revealed a significant association between *Blastocystis* ST1 infection and nausea (OR = 11.200, 95% CI: 1.643–17.634, *P* = 0.038) and *Blastocystis* ST3 infection and flatulence (OR = 12.286, 95% CI: 4.293–20.161, *P* < 0.001).

## Discussion

Although *Blastocystis* is the most common protist isolated in an Aboriginal community in Malaysia [12, 21, 22], to date, no epidemiological study has been carried out to investigate the distribution and associated risk factors of *Blastocystis* subtypes in relation to seasonal variation.

The present study successfully isolated *Blastocystis* ST1, ST2, ST3 and ST4 from the Aboriginal communities with an overall prevalence of 40.2%, where 42.6 and 37.8% of the subjects were infected with *Blastocystis* ST1-ST4 during the wet and dry seasons, respectively. Phylogenetic analysis confirmed subtype assignation with moderate to high bootstrap values, which grouped each *Blastocystis* subtype into the clades representing ST1 to ST4. The branching order obtained following phylogenetic analysis agreed with the proposed subtype order by Stensvold et al. [23] based on the consensus of previous studies [1, 17].

*Blastocystis* ST1, ST2, ST3 and ST4 are commonly found in humans [24–26] and account for nearly 90% of all human *Blastocystis* in parasitological surveys which performed genotyping [27]. The differences in the prevalence rates of ST1, ST2, ST3 and ST4, observed in this present study suggests different dynamic transmission may exist between the subtypes. These could include differences in host susceptibility, cyst numbers excreted by the host, cyst survival in host and environment, exposure of the host to the infective cyst, infectious dose ingested by the host and other elements of transmission cycle involved.

Distribution of *Blastocystis* subtypes differed geographically; the higher prevalence rate of ST3 concurs with other studies [28–30]. Similar observations were also reported in studies carried out among schoolchildren in Malaysia. Nithyamathi et al. [22] reported high prevalence rates of ST3, followed by ST1, ST2, ST4, and ST5. A study by Abdulsalam [31] observed 39.4, 36.4 and 18.2% of the rural schoolchildren were infected with ST3, ST1 and ST2, respectively. Nonetheless, a study among school children in Thailand showed ST1 (77.9%) was the most common subtype followed by ST2 (22.1%) [32]. A study in Ilero, Nigeria found ST1 as the most prevalent subtype followed by ST2 and ST3 [14].

Except for *Blastocystis* ST1, the prevalence rates of *Blastocystis* ST2, ST3 and ST4 infections did not indicate a significant difference between the wet and dry seasons. Nevertheless, descriptive analysis shows that the prevalences of *Blastocystis* ST2 (wet = 6.6%, dry = 4.6%) and ST4 (wet = 0.8%, dry = 0.5%) were observed to be low and unchanged throughout the two seasons while the prevalence of ST3 was persistently high throughout both seasons (wet = 18.8%, dry = 23.0%). *Blastocystis* ST3 is probably the only genotype of human origin, and this idea is supported by few studies carried out in urbanised cities state such as in Japan and Singapore, where *Blastocystis* ST3 was predominantly isolated and zoonotic transmission was rare [33, 34]. High prevalence of ST3 encountered in this present study during both seasons may be explained by the role of humans as a source of infection of *Blastocystis* ST3. The mode of transmission of *Blastocystis* ST3 infection remains unclear. It can be postulated that infection occurred within the family members; direct transmission from person to person is the possible mode of transmission of *Blastocystis* ST3. However, indirect transmission through ingestion of cysts present in water or food contaminated with faeces cannot be excluded. This is in agreement with a study by Yoshikawa et al. [35] which confirmed that *Blastocystis* ST3 was transmitted from human to human in two health care facilities in Osaka. Autoinfection has also been thought to be responsible in sustaining the infection in the host [36]. All these factors may be responsible for maintaining the transmission cycle within the family.

Although there was a significant low prevalence of *Blastocystis* ST1 infection in the dry season in comparison to the wet season, the presence of other family members with ST1 infection was persistently a significant factor to contract *Blastocystis* ST1 infection during both seasons. This shows that living with family members with ST1 infection, sharing facilities and having close contact with the family members may transmit the infections to at least one of the family members and sustain the transmission cycle. Human to human transmission of *Blastocystis* ST1 have been reported in Bangkok [13]. The present study adds to the statistic that person to person transmission of *Blastocystis* ST1 could occur. In addition, it can be concluded that seasonal variation has no role in the transmission cycle of *Blastocystis* ST1. Similarly, person to person transmission of *Blastocystis* ST2 occurred in this community for the Aborigines who have other family members infected with ST2 infection with the odds of 3.240 during the wet season and 5.559 during the dry season.

As stated above, the prevalence rate of all *Blastocystis* subtypes detected (ST1-ST4) in this present study was 42.6% in the wet season and 37.8% during the dry season, although these prevalences were not statistically different. This finding is in line with the report of a high number of cases of giardiasis and cryptosporidiosis seen during high rainfall months and low number of cases of both protozoan diseases during the dry season [37–39]. Both *Giardia* and *Cryptosporidium* were reported to be waterborne protozoa [40]. Since the pattern of seasonal variation in the prevalence of *Blastocystis* infection in this study was in agreement with the seasonal pattern of infection by *Giardia* and *Cryptosporidium*, we suspect that water would be one of the important factors in the dynamic transmission of *Blastocystis* in this community.

The present study identified use of stored river water for domestic activities as another strong risk factor that was significantly associated with *Blastocystis* infection during both seasons. When analysed further for each subtype, this risk factor is greater for *Blastocystis* ST3 infection during wet and dry seasons. The practice of drinking unboiled collected and stored river water could explain the high risk of *Blastocystis* infection as described by Anuar et al. [12]. This significant association was again supported by our study findings conducted in the same study areas which reported the occurrence of *Blastocystis* ST3 in all seven water samples collected during the wet and dry season from two rivers used by the communities [41]. The river water might be contaminated with human and animal faeces as open defecation are still being practised by almost 43.3% of the communities in the studied areas. Furthermore, the ST3 alleles identified in the river water samples during the wet and dry seasons were also detected in the Aborigines in this present study. During the wet season, ST3 allele 31, 34 and 38 were identified in the river water samples [41], and these alleles were identical to that of *Blastocystis* ST3 alleles in the human faecal samples. Similarly, in the dry season, the ST3 alleles detected in the human faecal samples were allele 34 and 38, and these particular alleles were also identified in the river water samples.

This present study also reported that the risk of getting an ST3 infection is higher during the dry season (OR = 8.069) compared to wet season (OR = 2.418) with the use of stored river water. This may be attributed to the use of more river water during the dry season as other resources of untreated water are delimited. Water from rivers and streams was important sources of routine activities by Aborigines residing in the villages located at the most upstream and midstream. These villages were not equipped with treated tap water supply as compared to more structured villages in the downstream. Water from rivers was either used directly or stored in containers and used during a shortage of tap water supply.

The role of water as a source of infection has been suggested by some epidemiological studies; Li et al. [42] identified that infection caused by *Blastocystis* ST1 was associated with consumption of raw water plants. *Blastocystis* ST1 has been reported by Abdulsalam [31] to be genetically identical in water samples and faeces of schoolchildren. Drinking water and river water contains *Blastocystis* ST1 which was also found in humans [32, 43]. This present study did not support that water is a source of ST1 infection, although similar allele of ST1 (allele 4) was identified in both water and faecal samples of the Aborigines during wet and dry seasons. In addition, the prevalence of *Blastocystis* ST1 in humans which were significantly lower during the dry season coincides with the absence of *Blastocystis* ST1 in the river water samples during the dry season [39]. Therefore, we hypothesise that river water is one of the sources of *Blastocystis* ST1 infection in this community.

Despite the studies on the waterborne potential of *Blastocystis* ST1, to date, there are a lack of studies which report the waterborne potential of *Blastocystis* ST3. The exception is the study by Li et al. [42], which reported an association between *Blastocystis* ST3 infection and drinking unboiled water. In Malaysia, Suresh et al. [44] identified *Blastocystis* ST3 in rivers. However, there is no information on the isolation of similar subtype in humans. We believe this study is the first to report waterborne transmission of *Blastocystis* ST3 in the Aboriginal community in Malaysia in relation to seasonal variation. Besides that, similar to the findings of ST1, there was no significant risk factor related to the usage of river water and *Blastocystis* ST2 infection. However, we identified ST2 allele 15 in the water samples from our previous study [41], and the same subtype and allele were also detected in the human faecal samples during the wet season. Waterborne transmission of blastocystosis is not unexpected as *Blastocystis* cysts can survive in different types of water; this is because cysts excreted to the external environment from hosts have a thick wall which confers survival advantage of the cyst outside the hosts [45].

Monthly household income of less and equal to RM 500 was found to be a significant risk factor for *Blastocystis* infection during the dry season. This finding was in agreement with the study by Nithyamathi et al. [22] which indicated that school children with low household income had two times higher risk to contract blastocystosis. Lower family income was correlated with the higher prevalence of intestinal parasitic infections [46]. Low income resulted in poor outcomes including poor nutrition, poor medical care and increase exposure to noxious agents. Low socioeconomic status has also been strikingly associated with high rates of infectious and parasitic diseases [47]. It is not surprising that low monthly household income in this present study was significantly associated with *Blastocystis* ST3 infection during the dry season. Most of the Aborigines work as rubber tappers and farmers. The worst heavy flood in decades hits the north-eastern part of Malaysia in end of December 2014 until early of January 2015 [48], where the study area was also affected. The collection of faecal samples during the wet season was performed in October to November 2014 (approximately 2 months before the heavy flood) and during the dry season, which was in June 2015 (approximately 5 months after the heavy flood). This flood gave an adverse effect to part of the economy, especially in the agricultural sector. Rubber prices were affected where the flood has disrupted supplies from Malaysia to other countries [48]. Therefore, since the Aborigines mostly earned daily income from rubber tapping and farming, their income was highly affected by the flood until a few months after the natural disaster. The low income may contribute to poor nutrition, sanitation and medical conditions and indirectly affect the health status of communities. All of the above descriptions may explain the finding that the risk of contracting *Blastocystis* ST3 infection was two times higher during the dry season among Aborigines who gained a monthly household income of less or equal to RM 500.

This present study failed to show any association of *Blastocystis* ST1 infection with age groups and occupation by multivariate model. Similar findings were also observed in *Blastocystis* ST2 infection where there was a significant association between *Blastocystis* ST2 infection and age groups, gender and not washing hands after having contact with soil using univariate model but not significant by logistic regression model. These findings contradicted previously reported a significant association between *Blastocystis* ST1 infection in patients with median age of 27 years old [49]. In another study, the prevalence of *Blastocystis* ST1 was significantly higher in patients aged 15–50 years old [50]. Based on gender, previous studies reported a higher prevalence of *Blastocystis* sp. infection in male in comparison to female [51, 52].

Clinical presentations of *Blastocystis* infection include non-specific gastrointestinal symptoms including diarrhoea, abdominal pain, flatulence and nausea [53, 54]. Among the gastrointestinal symptoms that were previously reported commonly associated with *Blastocystis* sp. infection is excessive flatulence with occasional diarrhoea and abdominal cramps [55]. It has been suggested that the symptoms associated with the infection are subtype-related. Among other subtypes detected, *Blastocystis* ST1 has been proven clinically and statistically to have the pathogenic potential [56, 57]. Yan et al. [58] suggested a possible relationship between *Blastocystis* ST1 infection and gastrointestinal symptoms. In this present study, *Blastocystis* ST1 infection without other intestinal parasitic infections was found to be significantly associated with nausea among the Aborigines. El Safadi et al. [59] reported a significant association between *Blastocystis* ST1 infection and gastrointestinal symptoms, where the most common symptom observed in the symptomatic individuals was diarrhoea. However, no published study has been previously associated ST1 with nausea, although nausea has been attributed as one of the non-specific symptoms of *Blastocystis* infections [4].

Flatulence was among the most prominent symptoms and significantly observed more common in individuals positive for blastocystosis without other intestinal parasitic infections than *Blastocystis*-infected individuals co-infected with other intestinal parasites [60]. This present study revealed a significant association of single infection of *Blastocystis* ST3 infection and flatulence and this confirmed findings reported by Tan et al. [61] and Souppart et al. [62]. This present study adds on the highlights of the pathogenic potential of *Blastocystis* ST1 in causing gastrointestinal symptoms particularly nausea in the infected individuals and *Blastocystis* ST3 in causing flatulence.

It is known that genetic heterogeneity existed among *Blastocystis* isolated from humans. The most diverse subtype among all studied subtype is probably *Blastocystis* ST3 [63]. In this present study, we identified heterogeneity in ST3 with allele 31, 34 and 38 and nucleotide diversity within subtypes were also found in ST2 with allele 15 and 131 (Table 3). Although genetic diversity was identified, however, since phylogenetic tree showed all *Blastocystis* subtypes were grouped under similar clades, therefore it is postulated that nucleotide sequence within ST2 and ST3 in this study are only minimally different.

**Table 3:** *Blastocystis* detected in the human faecal samples collected

Sample code	Subtype	Accession number	18S rRNA allele
Wet season			
T16	1	KX351975	4
LW95	1	KX351976	4
LW7	1	KX351977	4
P124	1	KX351978	4
P31	2	KX351979	15
LW161	2	KX351980	15
P252	2	KX351981	15
T22	3	KX351982	38
P39	3	KX351983	38
T30	3	KX351984	38
LW45	3	KX351985	31
P72	3	KX351986	38
T19	4	KX351987	94
Dry season			
KTA 58	1	KX351988	4
KPA 25	1	KX351989	4
LWA 50	1	KX351990	4
LWA 20	2	KX351991	131
LWA 116	2	KX351992	15
KPA 45	3	KX351993	34
KTA 08	3	KX351994	34
KPA 129	3	KX351995	34
LWA 87	3	KX351996	34
KPA 183	4	KX351997	42

## Conclusions

In conclusion, this present study is the first study that provides new insights into the distribution and risk factors of *Blastocystis* subtypes infections during wet and dry seasons among Aboriginal communities in Pahang, Malaysia. *Blastocystis* ST1, ST2, ST3 and ST4 were successfully isolated from the subjects during both seasons; high prevalence of *Blastocystis* ST3 was observed throughout the two seasons. The findings of this present study confirmed that presence of other family members infected with *Blastocystis* ST1, ST2 and ST3 was the significant risk factors associated with each distinctive *Blastocystis* subtype infection during wet and dry seasons. Infection occurs within family members and infected family members served as the source of infections of *Blastocystis* ST1, ST2 and ST3, thus this indicated person to person transmission. In addition to that, usage of stored river water for domestic activities was the significant risk factors for the acquisition of *Blastocystis* ST3 infection during wet and dry seasons. Therefore, besides person to person transmission, another mode of transmission of ST3 in this community was through contaminated collected stored river water. Pathogenic potential of *Blastocystis* ST1 and ST3 in the present study may highlight the need to relate gastrointestinal symptoms with subtypes in future studies.

## Additional file

Additional file 1: Table S1. Univariate analysis of the potential risk factors associated with *Blastocystis* subtype infections among the Aborigines in Temerloh, Pahang, Malaysia during the wet and dry seasons. (DOCX 29 kb)
