# Supplementary material for: Molecular epidemiology of blastocystosis in Malaysia: does seasonal variation play an important role in determining the distribution and risk factors of Blastocystis subtype infections in the Aboriginal community?
Source: Parasit Vectors. 2017 Jul 31;10:360. doi: 10.1186/s13071-017-2294-2 (PMC5537991; doi:10.1186/s13071-017-2294-2)
Supplement: Additional file 1: Table S1. — Univariate analysis of the potential risk factors associated with Blastocystis subtype infections among the Aborigines in Temerloh, Pahang, Malaysia during the wet and dry seasons. (DOCX 29 kb) [file 13071_2017_2294_MOESM1_ESM.docx]

**Additional file 1: Table S1** Univariate analysis of the potential risk factors associated with *Blastocystis* subtype infections among the Aborigines in Temerloh, Pahang, Malaysia during the wet and dry seasons

| **Variables** | **Infected (%)** | | **OR (95% CI)** | | ***P*-value** | |
| --- | --- | --- | --- | --- | --- | --- |
|  | **Wet season** | **Dry season** | **Wet season** | **Dry season** | **Wet season** | **Dry season** |
| Age |  |  |  |  |  |  |
| ST1 (≤15) | 12/256 (4.7) | 8/217 (3.7) | 0.431 (0.210-0.887) | 0.725 (0.288-1.827) | 0.020^*^ | 0.494 |
| ST1 (>15) | 30/256 (11.7) | 13/217 (6.0) | 1 | 1 |  |  |
| ST2 (≤15) | 12/256 (4.7) | 3/217 (1.4) | 3.169 (1.082-9.278) | 0.505 (0.127-2.008) | 0.028^*^ | 0.324 |
| ST2 (>15) | 5/256 (2.0) | 7/217 (3.2) | 1 | 1 |  |  |
| ST3 (≤15) | 22/256 (8.6) | 21/217 (9.7) | 1.046 (0.557-1.965) | 0.846 (0.447-1.603) | 0.888 | 0.609 |
| ST3 (>15) | 26/256 (10.2) | 29/217 (13.4) | 1 | 1 |  |  |
| ST4 (≤15) | 2/256 (0.8) | 0/217 (0.0) | - | - | - | - |
| ST4 (>15) | 0/256 (0.0) | 1/217 (0.5) | - | - |  |  |
| Gender |  |  |  |  |  |  |
| ST1 (Female) | 21/256 (8.2) | 7/217 (3.2) | 0.814 (0.420-1.577) | 0.490 (0.190-1.266) | 0.541 | 0.135 |
| ST1 (Male) | 21/256 (8.2) | 14/217 (6.5) | 1 | 1 |  |  |
| ST2 (Female) | 5/256 (2.0) | 5/217 (2.3) | 1.326 (1.112-2.956) | 1.050 (0.295-3.734) | 0.033^*^ | 0.941 |
| ST2 (Male) | 12/256 (4.7) | 5/217 (2.3) | 1 | 1 |  |  |
| ST3 (Female) | 31/256 (12.1) | 29/217 (13.4) | 1.688 (0.881-3.238) | 1.614 (0.852-3.057) | 0.112 | 0.140 |
| ST3 (Male) | 17/256 (6.6) | 21/217 (9.7) | 1 | 1 |  |  |
| ST4 (Female) | 1/256 (0.4) | 0/217 (0.0) | 0.841 (0.052-13.587) | - | 0.903 | - |
| ST4 (Male) | 1/256 (0.4) | 1/217 (0.5) | 1 | - |  |  |
| Total household members |  |  |  |  |  |  |
| ST1 (≥8) | 29/256 (11.3) | 6/217 (2.8) | 1.279 (0.628-2.605) | 0.556 (0.207-1.494) | 0.496 | 0.239 |
| ST1 (<8) | 13/256 (5.1) | 15/217 (6.9) | 1 | 1 |  |  |
| ST2 (≥8) | 11/256 (4.3) | 4/217 (1.8) | 1.012 (0.361-2.833) | 0.976 (0.267-3.565) | 0.982 | 0.971 |
| ST2 (<8) | 6/256 (2.3) | 6/217 (2.8) | 1 | 1 |  |  |
| ST3 (≥8) | 32/256 (12.5) | 19/217 (8.9) | 1.128 (0.581-2.190) | 0.871 (0.455-1.666) | 0.722 | 0.675 |
| ST3 (<8) | 16/256 (6.3) | 31/217 (14.3) | 1 | 1 |  |  |
| ST4 (≥8) | 0/256 (0.0) | 1/217 (0.5) | - | - | - | - |
| ST4 (<8) | 2/256 (0.8) | 0/217 (0.0) | - | - |  |  |
| The presence of other family members with same *Blastocystis* subtype infection |  |  |  |  |  |  |
| ST1 (Yes) | 20/256 (7.8) | 6/217 (2.8) | 5.170 (2.535-10.544) | 4.500 (1.534-13.198) | <0.001^*^ | 0.003^**^ |
| ST1 (No) | 22/256 (8.6) | 16/217 (7.4) | 1 | 1 |  |  |
| ST2 (Yes) | 4/256 (1.6) | 4/217 (1.8) | 3.077 (1.057-8.545) | 2.688 (1.018-9.770) | <0.001^**^ | 0.001^**^ |
| ST2 (No) | 13/256 (5.1) | 2/217 (0.9) | 1 | 1 |  |  |
| ST3 (Yes) | 20/256 (7.8) | 31/217 (14.3) | 5.229 (2.571-10.634) | 13.021 (6.658-18.628) | 0.001^**^ | <0.001^**^ |
| ST3 (No) | 28/256 (10.9) | 21/217 (9.7) | 1 | 1 |  |  |
| ST4 (Yes) | - | - | - | - | - | - |
| ST4 (No) | - | - | - | - |  |  |
| Household income |  |  |  |  |  |  |
| ST1 (≤RM 500) | 29/256 (11.3) | 15/217 (6.9) | 0.973 (0.476-1.992) | 1.484 (0.551-3.993) | 0.941 | 0.432 |
| ST1 (<RM500) | 13/256 (5.1) | 6/217 (2.8) | 1 | 1 |  |  |
| ST2 (≤RM 500) | 11/256 (4.3) | 6/217 (2.8) | 0.790 (0.282-2.219) | 0.852 (0.233-3.116) | 0.655 | 0.809 |
| ST2 (<RM500) | 6/256 (2.3) | 4/217 (1.8) | 1 | 1 |  |  |
| ST3 (≤RM 500) | 34/256 (13.3) | 38/217 (17.5) | 1.079 (0.542-2.149) | 2.827 (1.656-7.765) | 0.828 | 0.029^*^ |
| ST3 (<RM500) | 14/256 (5.5) | 12/217 (5.5) | 1 | 1 |  |  |
| ST4 (≤RM 500) | 2/256 (0.8) | 0/217 (0.0) | - | - | - | - |
| ST4 (<RM500) | 0/256 (0.0) | 1/217 (0.5) | - | - |  |  |
| Occupation |  |  |  |  |  |  |
| ST1 (Rubber tapper, farmer) | 38/256 (14.8) | 14/217 (6.5) | 1.181 (0.043-0.755) | 1.063 (0.409-2.758) | 0.009^**^ | 0.901 |
| ST1 (Professional, others) | 4/256 (1.6) | 7/217 (3.2) | 1 | 1 |  |  |
| ST2 (Rubber tapper, farmer) | 17/256 (6.6) | 6/217 (2.8) | - | 0.783 (0.214-2.866) | - | 0.711 |
| ST2 (Professional, others) | 0/256 (0.0) | 4/217 (1.8) | - | 1 |  |  |
| ST3 (Rubber tapper, farmer) | 48/256 (18.8) | 38/217 (17.5) | - | 1.918 (0.933-3.943) | - | 0.073 |
| ST3 (Professional, others) | 0/256 (0.0) | 12/217 (5.5) | - | 1 |  |  |
| ST4 (Rubber tapper, farmer) | 2/256 (0.8) | 1/217 (0.5) | - | - | - | - |
| ST4 (Professional, others) | 0/256 (0.0) | 0/217 (0.0) | - | - |  |  |
| Water supply |  |  |  |  |  |  |
| ST1 (Untreated) | 27/256 (10.5) | 15/217 (6.9) | 0.971 (0.487-1.938) | 1.583 (0.589-4.259) | 0.934 | 0.359 |
| ST1 (Treated) | 15/256 (5.9) | 6/217 (2.8) | 1 | 1 |  |  |
| ST2 (Untreated) | 10/256 (3.9) | 7/217 (3.2) | 0.760 (0.279-2.070) | 1.440 (0.362-5.732) | 0.591 | 0.603 |
| ST2 (Treated) | 7/256 (2.7) | 3/217 (1.4) | 1 | 1 |  |  |
| ST3 (Untreated) | 40/256 (15.6) | 35/217 (16.1) | 3.254 (1.450-7.303) | 1.563 (0.792-3.084) | 0.003^**^ | 0.195 |
| ST3 (Treated) | 8/256 (3.1) | 15/217 (6.9) | 1 | 1 |  |  |
| ST4 (Untreated) | 2/256 (0.8) | 0/217 (0.0) | - | - | - | - |
| ST4 (Treated) | 0/256 (0.0) | 1/217 (0.5) | - | - |  |  |
| Source of washing |  |  |  |  |  |  |
| ST1 (Untreated) | 27/256 (10.5) | 15/217 (6.9) | 1.032 (0.518-2.058) | 1.583 (0.589-4.259) | 0.928 | 0.359 |
| ST1 (Treated) | 15/256 (5.9) | 6/217 (2.8) | 1 | 1 |  |  |
| ST2 (Untreated) | 10/256 (3.9) | 7/217 (3.2) | 0.803 (0.295-2.186) | 1.440 (0.362-5.732) | 0.667 | 0.603 |
| ST2 (Treated) | 7/256 (2.7) | 3/217 (1.4) | 1 | 1 |  |  |
| ST3 (Untreated) | 38/256 (14.8) | 35/217 (16.1) | 2.523 (1.921-5.341) | 1.563 (0.792-3.084) | 0.013^*^ | 0.195 |
| ST3 (Treated) | 10/256 (3.9) | 15/217 (6.9) | 1 | 1 |  |  |
| ST4 (Untreated) | 2/256 (0.8) | 0/217 (0.0) | - | - | - | - |
| ST4 (Treated) | 0/256 (0.0) | 1/217 (0.5) | - | - |  |  |
| Usage of stored river water |  |  |  |  |  |  |
| ST1 (Yes) | 20/256 (7.8) | 8/217 (3.7) | 0.980 (0.505-1.900) | 0.804 (0.319-2.026) | 0.952 | 0.643 |
| ST1 (No) | 22/256 (8.6) | 13/217 (6.0) | 1 | 1 |  |  |
| ST2 (Yes) | 11/256 (4.3) | 7/217 (3.2) | 2.079 (0.745-5.804) | 3.283 (0.826-13.055) | 0.155 | 0.076 |
| ST2 (No) | 6/256 (2.3) | 3/217 (1.4) | 1 | 1 |  |  |
| ST3 (Yes) | 32/256 (12.5) | 36/217 (16.6) | 2.571 (1.329-4.974) | 4.962 (2.476-9.946) | 0.004^**^ | <0.001^**^ |
| ST3 (No) | 16/256 (6.3) | 14/217 (6.5) | 1 | 1 |  |  |
| ST4 (Yes) | 1/256 (0.4) | 1/217 (0.5) | 1.082 (0.067-17.488) | - | 0.956 | - |
| ST4 (No) | 1/256 (0.4) | 0/217 (0.0) | 1 | - |  |  |
| Latrine system |  |  |  |  |  |  |
| ST1(No proper latrine system) | 20/256 (7.8) | 12/217 (5.5) | 1.407 (0.724-2.735) | 1.603 (0.646-3.978) | 0.313 | 0.306 |
| ST1 (Flush & pit latrine) | 22/256 (8.6) | 9/217 (4.1) | 1 | 1 |  |  |
| ST2 (No proper latrine system) | 7/256 (2.7) | 4/217 (1.8) | 1.025 (0.377-2.785) | 0.756 (0.207-2.758) | 0.962 | 0.671 |
| ST2 (Flush & pit latrine) | 10/256 (3.9) | 6/217 (2.8) | 1 | 1 |  |  |
| ST3 (No proper latrine system) | 25/256 (9.8) | 29/217 (13.4) | 1.775 (0.944-3.338) | 1.822 (0.961-3.454) | 0.073 | 0.064 |
| ST3 (Flush & pit latrine) | 23/256 (9.0) | 21/217 (9.7) | 1 | 1 |  |  |
| ST4 (No proper latrine system) | 1/256 (0.4) | 0/217 (0.0) | 1.466 (0.091-23.704) | - | 0.786 | - |
| ST4 (Flush & pit latrine) | 1/256 (0.4) | 1/217 (0.5) | 1 | - |  |  |
| Not washing hands after having contact with soil |  |  |  |  |  |  |
| ST1 (Yes) | 2/256 (0.8) | 1/217 (0.5) | 0.332 (0.076-1.451) | 0.526 (0.066-4.168) | 0.125 | 0.537 |
| ST1 (No) | 40/256 (15.6) | 20/217 (9.2) | 1 | 1 |  |  |
| ST2 (Yes) | 5/256 (2.0) | 1/217 (0.5) | 3.567 (1.161-10.959) | 1.242 (0.148-10.395) | 0.019^*^ | 0.841 |
| ST2 (No) | 12/256 (4.7) | 9/217 (4.1) | 1 | 1 |  |  |
| ST3 (Yes) | 9/256 (3.5) | 7/217 (3.2) | 2.055 (0.875-4.826) | 2.309 (0.844-6.313) | 0.093 | 0.095 |
| ST3 (No) | 39/256 (15.3) | 43/217 (19.8) | 1 | 1 |  |  |
| ST4 (Yes) | 0/256 (0.0) | 0/217 (0.0) | - | - | - | - |
| ST4 (No) | 2/256 (0.8) | 1/217 (0.5) | - | - |  |  |
| Did not wash hand after defaecation |  |  |  |  |  |  |
| ST1 (Yes) | 1/256 (0.4) | 1/217 (0.5) | 0.348 (0.045-2.724) | 1.175 (0.140-9.881) | 0.294 | 0.882 |
| ST1 (No) | 41/256 (16.0) | 20/217 (9.2) | 1 | 1 |  |  |
| ST2 (Yes) | 2/256 (0.8) | 1/217 (0.5) | 2.318 (0.479-11.228) | 2.764 (0.311-24.537) | 0.283 | 0.342 |
| ST2 (No) | 15/256 (5.9) | 9/217 (4.1) | 1 | 1 |  |  |
| ST3 (Yes) | 6/256 (2.3) | 2/217 (0.9) | 3.159 (1.067-9.351) | 0.952 (0.191-4.737) | 0.030^*^ | 0.952 |
| ST3 (No) | 42/256 (16.4) | 48/217 (22.1) | 1 | 1 |  |  |
| ST4 (Yes) | 0/256 (0.0) | 0/217 (0.0) | - | - | - | - |
| ST4 (No) | 2/256 (0.8) | 1/217 (0.5) | - | - |  |  |
| Presence of animals |  |  |  |  |  |  |
| ST1 (Yes) | 27/256 (10.5) | 13/217 (6.0) | 1.410 (0.710-2.801) | 1.145 (0.454-2.888) | 0.325 | 0.775 |
| ST1 (No) | 15/256 (5.9) | 8/217 (9.0) | 1 | 1 |  |  |
| ST2 (Yes) | 10/256 (3.9) | 7/217 (3.2) | 1.064 (0.392-2.889) | 1.658 (0.417-6.595) | 0.904 | 0.468 |
| ST2 (No) | 7/256 (2.7) | 3/217 (1.4) | 1 | 1 |  |  |
| ST3 (Yes) | 30/256 (11.7) | 31/217 (14.3) | 1.296 (0.680-2.472) | 1.177 (0.616-2.252) | 0.430 | 0.621 |
| ST3 (No) | 18/256 (7.0) | 19/217 (8.8) | 1 | 1 |  |  |
| ST4 (Yes) | 0/256 (0.0) | 1/217 (0.5) | - | - | - | - |
| ST4 (No) | 2/256 (0.8) | 0/217 (0.0) | - | - |  |  |
| The level of father’s education |  |  |  |  |  |  |
| ST1 (No formal education) | 15/202 (7.4) | 12/164 (7.3) | 1.973 (0.913-4.267) | 0.354 (0.076-1.645) | 0.081 | 0.169 |
| ST1 (Primary and secondary) | 16/202 (7.9) | 2/164 (1.2) | 1 | 1 |  |  |
| ST2 (No formal education) | 12/202 (5.9) | 6/164 (3.7) | 0.257 (0.056-1.180) | 0.367 (0.043-3.134) | 0.062 | 0.341 |
| ST2 (Primary and secondary) | 2/202 (1.0) | 1/164 (0.6) | 1 | 1 |  |  |
| ST3 (No formal education) | 24/202 (11.9) | 24/164 (14.6) | 1.045 (0.509-2.145) | 1.607 (0.756-3.416) | 0.904 | 0.215 |
| ST3 (Primary and secondary) | 15/202 (7.4) | 15/164 (9.1) | 1 | 1 |  |  |
| ST4 (No formal education) | 0/202 (0.0) | 0/164 (0.0) | - | - | - | - |
| ST4 (Primary and secondary) | 2/202 (1.0) | 0/164 (0.0) | - | - |  |  |
| The level of mother’s education |  |  |  |  |  |  |
| ST1 (No formal education) | 16/202 (7.9) | 12/164 (7.3) | 1.977 (0.912-4.288) | 0.429 (0.092-1.997) | 0.081 | 0.268 |
| ST1 (Primary and secondary) | 15/202 (7.4) | 2/164 (1.2) | 1 | 1 |  |  |
| ST2 (No formal education) | 12/202 (5.9) | 6/164 (0.6) | 0.294 (0.064-1.353) | 0.442 (0.052-3.778) | 0.097 | 0.444 |
| ST2 (Primary and secondary) | 2/202 (5.5) | 1/164 (0.6) | 1 | 1 |  |  |
| ST3 (No formal education) | 26/202 (12.9) | 26/164 (15.9) | 0.930 (0.444-1.948) | 1.516 (0.695-3.307) | 0.847 | 0.294 |
| ST3 (Primary and secondary) | 13/202 (6.4) | 13/164 (7.9) | 1 | 1 |  |  |
| ST4 (No formal education) | 2/202 (1.0) | 0/164 (0.0) | - | - | - | - |
| ST4 (Primary and secondary) | 0/202 (0.0) | 0/164 (0.0) | - | - |  |  |

^*^*P* < 0.05, ^**^*P* < 0.01
